# Leader Behavioral Integrity and Employee In-Role Performance: The Roles of Coworker Support and Job Autonomy

**DOI:** 10.3390/ijerph17124303

**Published:** 2020-06-16

**Authors:** Yongjun Choi, David J. Yoon, Dongkyu Kim

**Affiliations:** 1 College of Business Administration, Hongik University, Seoul 04066, Korea; 2 Department of Management and Marketing, Perdue School of Business, Salisbury University, Salisbury, MD 21801, USA; jeehyuny@yahoo.com; 3 Department of People and Organizations, NEOMA Business School, 76825 Mont-Saint-Aignan, France; dongkyu.kim@neoma-bs.fr

**Keywords:** behavioral integrity, in-role performance, coworker support, job autonomy

## Abstract

The positive relationship between leader behavioral integrity and an employee’s in-role performance is well-established, but explanations for why this effect exists are still in a nascent stage. Drawing upon leader behavioral integrity theory and job-demands resources theory, the authors explain how leader behavioral integrity facilitates employee in-role performance and the boundary conditions influencing the relationship between leader behavioral integrity and employee in-role performance. Using multisource data from 209 employee-manager dyads in South Korea, this paper found support for the mediating effect of coworker support in the positive relationship between leader behavior integrity and employees’ in-role autonomy. Furthermore, compared to those who perceive low job autonomy, the positive indirect effect of leader behavioral integrity on in-role performance via coworker support was stronger for employees who perceive high job autonomy. The findings emphasize the importance of a leader’s individual difference (i.e., leader behavioral integrity) and job resources (i.e., job autonomy) facilitating the receipt of team members’ supporting behaviors which, in turn, energize employee in-role performance. Theoretical and practical implications are discussed.

## 1. Introduction

Employee vitality is integral for sustainable employee performance at work. Consequently, to build healthier and more productive workforce in dynamic and challenging business environments, it is crucial for leaders to ensure and even stimulate employee vitality [1]. For sustainable growth and viability of organizations, more specifically to stimulate employee viability, a growing body of research calls for leaders with integrity [2]. However, although the special issue *of International Journal of Environmental Research and Public Health* about “The vital worker: Towards sustainable performance at work” in 2019 greatly extended the current understanding of vital employees and sustainable performance in organizations, relatively little attention has been paid to the roles of leaders, especially the leaders with integrity.

Leadership researchers have long emphasized the role of integrity as an integral trait for being an effective leader by touting its numerous advantages at work [3,4,5,6,7,8,9]. The seminal works by Simons [7,8] on leader behavioral integrity, “the perceived alignment between a leader’s words and deeds” [8] (p. 18), have further sparked empirical studies on leader behavioral integrity in the last two decades. Specifically, the literature on leader behavioral integrity argues that, while holding the implicit assumption that the words spoken by a leader are ethical [10], leader behavioral integrity is distinct from other leadership styles in that it represents a trait ascribed to a leader [8,11,12,13].

The positive relationship between leader behavioral integrity and an employee’s in-role performance is well established in the literature [11,13,14,15,16,17,18,19]. Accordingly, scholars have explored why, or through what mechanisms, this relationship exists. The literature on leader behavioral integrity showed that trust and role clarity can explain the mechanism. For example, analogous to Simons’s [8] proposition, previous studies showed that leader behavioral integrity increases employees’ in-role performance because it fosters employees’ trust in their supervisor which, in turn, energizes their in-role performance (e.g., [12,14]). Specifically, trust in leadership serves as a means to increase the predictability and reliability of leader behaviors (e.g., [14,16,18]). Indeed, a meta-analysis also provides support that the positive effects of leader behavioral integrity on in-role performance is largely mediated by employees’ trust in leadership [13]. In addition to the trust in leadership, previous studies showed that, because employees who perceive that their supervisors have high integrity also experience much clearer communication with their supervisors [16] and also because these employees better understand what is expected of them [14], they are more likely to show higher in-role performance. However, the current understanding of how leader behavioral integrity affects employees’ in-role performance is still in a nascent stage; researchers, therefore, called for further investigation of the underlying mechanisms [20,21]. For example, employees may gain advantages when their leader demonstrates behavioral integrity and this advantage may help account for the process through which leader behavioral integrity leads to high in-role performance.

In addition, previous studies demonstrated that the effects of leader behavioral integrity on its outcomes can vary depending on contextual factors. For example, Simons and Hagen [22] showed that behavioral integrity leads to trust when the trustee has more power than the trustor. Tomlinson et al. [15] also showed that leader behavioral integrity and the value congruence between an employee and manager interact in predicting one’s organizational identity. That is, whereas the positive effect of leader behavioral integrity on organizational identity remains relatively stable when value congruence is high, when value congruence is low, it lessens the positive effect of leader behavioral integrity on organizational identity. However, a dearth of research has examined the boundary conditions that influence the relationship between leader behavioral integrity and in-role performance; thus, researchers called for empirical research of moderators related to employees’ perceptions of their context [23]. 

To address this research gap, this current study aims to elucidate the relationship between leader behavioral integrity and in-role performance. To do so, the present study draws upon the job demands–resources (JD-R) model to develop the theoretical framework. Specifically, the present study focuses on the motivational processes that are initiated by job resources [24,25,26], especially coworker support and perceived job autonomy. Central to the theory of this current study is the mediating role of coworker support in the leader behavioral integrity and in-role performance link along with the moderating effect of job autonomy in this mediated relationship. 

This study extends current literatures in several ways. First, this paper contributes to the research on behavioral integrity by introducing another type of mediator, coworker support, in the behavioral integrity and in-role performance link. Although behavioral integrity can affect a multitude of employees whom the leader interacts with at work, empirical findings have been limited to a single employee’s perceptions toward a leader and his or her work attitude and behaviors. Accordingly, relatively less is known about how coworkers’ behaviors explain the relationship between leader behavioral integrity and employees’ in-role performance. This current study examines whether leader behavioral integrity can facilitate coworker support which, in turn, positively affect one’s in-role performance. Second, the present study contributes to the current literature on leader behavioral integrity by examining the moderating role of autonomy—an important job resource—in the relationship between leader behavioral integrity and employees’ in-role performance. Specifically, answering Prottas’s [27] call for further empirical research that examines the moderating role of autonomy in the relationship between leader behavioral integrity and employee outcomes, this paper introduces job autonomy as a potential moderator and investigate the importance of leader behavioral integrity when an employee perceives high job autonomy. To the best of the authors’ knowledge, no studies have examined how job autonomy enhances or weakens the impact of leader behavioral integrity on employee outcomes. As such, the present study tests whether leader behavioral integrity facilitates an employee’s in-role performance especially in a context where the employee perceives high job autonomy.

## 2. Materials and Methods

### 2.1. Hypotheses Development

#### 2.1.1. Coworker Support as a Mediator

The present study posits that coworker support can explain how leader behavioral integrity fosters an employee’s in-role performance. The JD-R model [25] suggests that every job has specific risk factors that can increase stress and these factors can be categorized into two categories with two different processes. The first process relates to job demands that employees experience on a daily basis. These job demands may be mental, emotional, or physical in nature (e.g., physical workload, time pressure, contact with customers, physical environment, shift work) [25] and depletes employees’ energy. This leads to strain and exhaustion. The second process relates to job resources. These job resources may be interpersonal, work, and task-related (e.g., proper supervisory feedback and support, good rewards, more job control and participation, higher job security) [25]. These job resources are integral in helping employees achieve their work goals and keep them from being disengaged at work [28,29,30]. Social support—including those from coworkers—is an important job resource because coworker support (1) increases the likelihood that an employee achieves work goals, which leads to engagement in their work role and (2) decreases the work demands as coworker support often reduces the time it takes for the employee to complete his or her prescribed tasks [28]. 

Leader behavioral integrity can increase the degree of support that employees receive from their coworkers. According to the JD-R model, supervisor’s support and proper feedback to his or her employees are an important way that employee replenishes their job resources [28]. A leader’s behavioral integrity closely relates to proper feedback—an important job resource—for two reasons. First of all, according to the uncertainty management perspective [31], a leader who demonstrates behavioral integrity reduces uncertainty among the members that he or she manages. In supervisory relationships, employees seek consistency of supervisory behaviors. When supervisors’ behaviors are different from their words, it (1) prevents the employees from forming a coherent self-evaluation (“Based on what my boss is saying, am I do my job well?”), (2) undermines employees’ ability to predict and exercise control of their work environment (“Based on what my boss is saying, I should go and fix these items”), and (3) shakes the employees’ sense of security in the workplace (“Should I interpret what my boss is saying to mean that I’ll be let go soon or that I’m doing ok?”) [31]. As such, for employees who have a supervisor with high behavioral integrity, their sense of relational uncertainty in the workplace—and the stress that it may create—reduces. This is consistent with the JD-R model [28] wherein a clear communication and feedback channel with one’s supervisor increases an employee’s job resources. This surplus of job resources allows employees to stay engaged in the workplace [28] and focus their attention on providing job resources to others to complete their tasks.

The positive effect of leader behavioral integrity on providing employees with job resources is not confined to a single leader–member dyad. Instead, leader behavioral integrity positively affects all employees that work under the leader. Although leader behavioral integrity has been mostly examined on the dyadic level (e.g., [12,14,16,30]), a growing body of research suggests that leader behavioral integrity affects most team members in a similar manner. That is, since employees within the same team are exposed to similar behaviors of the supervisor, they are likely to have similar perceptions of their leader’s behavioral integrity [15,32]. For example, Dineen et al. [15] showed that both individual and aggregate perceptions of leader behavioral integrity within the same team energize employees’ organizational citizenship behaviors (OCBs) because leader behavioral integrity makes employees consider OCBs as appropriate behaviors in their teams. In a similar vein, Leroy et al. [32] demonstrated in data from 54 nursing teams that nurses within the same team have similar perception of their leader behavioral integrity for safety. A meta-analysis also provides support that an employee who perceives high leader behavioral integrity is more likely to display extra-role behaviors, such as OCBs [13]. Thus, it is likely that employees in the same team are likely to perceive the same word-and-deed alignment of their supervisors and, thus, will be motivated to respond to the leader behavioral integrity by providing more support to their coworkers within their teams. Because leader behavioral integrity replenishes employees with job resources, employees have more job resources to expend on helping others [11,15,19,33]. 

Coworker support—a job resource at the interpersonal level [29]—plays an important role in energizing an employee’s positive work attitudes and behaviors. Previous studies suggest that employees’ helping behaviors toward their coworkers, such as organizational citizenship behaviors (OCBs), can capture the extent to which an employee perceives coworker support (e.g., [33,34,35,36,37]). In regard to work attitudes, studies showed that employees who perceive a high level of coworker support are likely to be more satisfied with their jobs and less likely to leave the organization [38,39]. Chiaburu and Harrison’s [40] meta-analysis also provides support that employees who perceive high coworker support show high job satisfaction and organizational commitment [40]. Regarding work behaviors, specifically an employee’s in-role performance, research shows that coworker support is important for increasing an employee’s in-role performance because it facilitates the internalization of work roles [41], providing employees with more resources [42] and task-directed helping [43], and supplementing the formal support provided by supervisors [44]. Indeed, a meta-analysis also showed that coworker support increases an individual’s in-role performance [40]. Taken together, this present study proposes that leader behavioral integrity enhances an employee’s in-role performance by that focal employee with more coworker support.

**Hypothesis** **1 (H1).** *The positive relationship between leader behavioral integrity and in-role performance is mediated by coworker support.*

#### 2.1.2. Job Autonomy as a Moderator

Up to this point, this study has suggested that behavioral integrity enhances the extent of coworker support that employees receive in their team and subsequently improves their in-role performance. The other side of the story is the characteristic of the job itself that allows employees in those jobs to fully take advantage of the consistent message that leaders provide in the form of behavioral integrity. One of the most prominent characteristics of a job that affects employees’ performance is the degree of autonomy that they have on their job [45,46,47,48]. According to Hackman and Oldham [45], job autonomy is “the degree to which the job provides substantial freedom, independence, and discretion to the individual in scheduling the work and in determining the procedures to be used in carrying it out” (p. 162). Under the JD-R model, having an abundance of job resource such as job autonomy motivates and engages employees [26,29,49,50], which can subsequently lead to their high in-role performance. Furthermore, job autonomy is critical in improving employees’ motivation and in reducing the effects of job demands on job strain [28]. Hence, among different types of job resources, job autonomy has shown to be the most important resource that employees require in completing their day-to-day tasks [51]. 

Because job autonomy encourages employees to seek out resources to better perform their jobs [52,53], the current study argues that the extent to which employees receive coworker support as a result of leader behavioral integrity may vary depending on their job autonomy. Employees who perceive high job autonomy are likely to feel self-controlled [54], enjoy their work [49], and be more engaged in and responsible for their jobs [30,55,56,57]. Since they have much more latitude at work, they are motivated to seek out resources necessary to better perform their jobs [58]. Previous studies showed that employees seek job-relevant resources from organizational insiders [59,60,61,62,63]; the most prominent example of organizational insiders that employee can find are their coworkers. Coworkers provide important job-relevant resources such as information on role demand as well as technical, and normative information; these types of information are beneficial for employees’ in-role performance [40,61,64]. When employees have a high degree of job autonomy, they have fewer restrictions in utilizing the job resources that they received from their supervisor in the form of consistent feedback and communication, which are marks of behavioral integrity. With an abundance of such job resources that the supervisor provides, employees with high job autonomy are more likely to more freely seek and receive help from their coworkers to find the best way to improve their performance, resulting in developing and utilizing positive coworker relationships [65,66,67,68].

In sum, the translation of leader behavioral integrity into coworker support will be enhanced as a result of employees’ perception of job autonomy, which, in turn, will increase their in-role performance. In low job autonomy situations, employees have little discretion to decide and/or carry out their jobs within a team. Hence, despite leader behavioral integrity, they are in less need of support and feedback from their coworkers because their tasks and behaviors are already prescribed [69]. As such, compared to those with high job autonomy, the translation of leader behavioral integrity into coworker support may be weakened.

**Hypothesis** **2 (H2).** *The strength of the mediated relationship between leader behavioral integrity and in-role performance (via coworker support) varies based on job autonomy; the indirect effect of leader behavioral integrity via coworker support on in-role performance will be stronger for those with high job autonomy than for those with low autonomy.*

**Figure 1:**
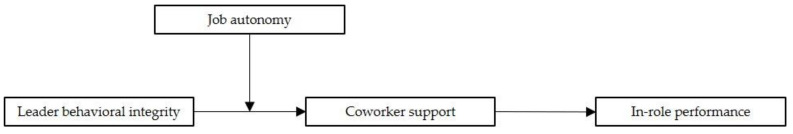
Conceptual model.

Our conceptual model is presented in Figure 1.

### 2.2. Methods

#### 2.2.1. Participants and Procedure

Pairs of questionnaire packages were randomly distributed to employees and their direct managers from 40 firms located in Republic of Korea (66.88% manufacturing, 16.1% public enterprises, 14.1% information technology services, 1.5% financial and insurance services, and 1.5% other service industries). The research team met 300 employees to inform them regarding the purpose of the current study and to describe the procedure for completing the survey during their free time. Employees completed the questionnaire including leader behavioral integrity, coworker support, job autonomy, leader–member exchange (LMX), and demographic information. After completing the questionnaire, they asked their managers to fill out the questionnaire assessing their in-role performance and demographic information. In order to avoid nesting issues in testing the hypothesized relationships, the research team recruited one employee-manager dyad from each team, so the sample consists of unique dyads. The research team-assigned identification number was encoded on each questionnaire to match the immediate manager’s evaluations with each follower’s responses. Both employees and managers sent the survey packets directly to the research team using a pre-addressed reply envelope. All participants were assured that their responses would remain confidential. In addition, all subjects gave their informed consent for inclusion before they voluntarily participated in the study. The study was conducted in accordance with the Declaration of Helsinki of 1975.

Among the 300 employee-manager dyads who received the survey, 227 were returned, giving a response rate of 75.67%. Eliminating surveys with missing responses, a final sample of 209 employee-supervisor dyads was used to test the hypotheses. The average age of employees was 30.39 years (SD = 4.57) and 76.6% of them were male. For managers, their average age was 38.88 years (SD = 5.96) and 94.3% of them were male. On average, their organizational tenure was 3.42 years (SD = 3.05) and 7.90 years (SD = 6.01). 

#### 2.2.2. Measures

Whereas all the survey items were originally in English, the study participants of this study were Korean. Therefore, following Brislin’s [70] method, all the survey measures were translated from English to Korean, and back to English.

Simons et al.’s [18] eight-item scale was used to measure leader behavioral integrity (α = 0.94). On a 7-point Likert scale ranging from 1 (strongly disagree) to 7 (strongly agree) employees evaluated the extent to which each statement described their supervisor’s word-deed alignment. Sample items include “My manager delivers on promises” and “My manager practices what he/she preaches.” 

Coworker support was measured using Tsui et al.’s [71] seven-item Likert scales (α = 0.85). Sample items include “My coworkers seem willing to listen to my problems” and “My coworkers and I have confidence in one another.” Responses were given on seven-point Likert scale (1 = strongly disagree to 7 = strongly agree).

An employee’s direct supervisor provided information about the focal employee’s in-role performance using Williams and Anderson’s [72] seven-item scale (α = 0.89). Sample items include “This employee fulfills responsibilities specified in job description” and “This employee meets formal performance requirements of the job.” Respondents answered on a seven-point Likert scale (1 = strongly disagree to 7 = strongly agree).

Breaugh’s [73] nine-item scale was used to measure job autonomy (α = 0.92). On a seven-point Likert scale ranging from 1 (strongly disagree) to 7 = (strongly disagree), employees evaluated the extent to which each statement described their supervisor’s word-deed alignment. Sample items include “I am free to choose the method(s) to use in carrying out my work” and “My job is such that I can decide when to do particular work activities.” 

Leader–member exchange (LMX) was controlled because it affects a direct manager’s ratings of subordinates’ in-role performance [74]. Specifically, LMX was measured using Scandura and Graen’s [75] seven-item scale (α = 0.89). Sample items include “How well do you feel that your immediate supervisor understands your problems and needs?” and “How well do you feel that your immediate supervisor recognizes your potential?”. Respondents answered on a seven-point Likert scale. Following the recommendations for the use of control variables in regression analysis [76,77], employees’ and their managers’ gender, age, and tenure were not controlled because none of them were significantly correlated with this current study variables. 

## 3. Results

### 3.1. Data Analysis 

Descriptive statistics and zero-order correlations for the variables are presented in Table 1 and results for the regression analyses are shown in Table 2. As shown in Table 1, consistent with the predictions, leader behavioral integrity is positive related to both coworker support (*r* = 0.55, *p* < 0.001) and in-role performance (*r* = 0.27, *p* < 0.001). In addition, coworker support is positively associated with in-role performance (*r* = 0.33, *p* < 0.001). Regarding job autonomy, the moderating variable in the hypothesized model, it is positively related to leader behavioral integrity (*r* = 0.33, *p* < 0.001) and coworker support (*r* = 0.29, *p* < 0.001), but it has no significant relationship with in-role performance (*r* = 0.02, *n.s.*). Table 2 presents hierarchical regression results. The dependent variables for Models 1–4 and 5 are coworker support and in-role performance, respectively. As predicted, leader behavioral integrity increases coworker support (*β =* 0.55, *p* < 0.001; Model 2), and coworker support increases in-role performance (*β =* 0.27, *p* < 0.001; Model 5). Specifically, when including the proposed mediator (i.e., coworker support) and dependent variable (i.e., in-role performance) in the same model, the effect of leader behavior integrity on in-role performance is not significant (*β =* 0.13, *n.s.*; Model 5) suggesting full mediation effect of coworker support in the relationship between leader behavioral integrity and in-role performance. The interaction term between leader behavioral integrity and job autonomy is significant (*β =* 0.13, *p* < 0.05; Model 4) suggesting the potential moderating effect of job autonomy in the relationship between leader behavioral integrity and in-role performance via coworker support. 

To test the hypotheses, SPSS Statistics Version 23 was used. To test the mediation (i.e., hypothesis 1) and moderated mediation hypotheses (i.e., hypothesis 2), Preacher et al.’s [78] regression-based approach was used to calculate the indirect effect of leader behavioral integrity on in-role performance. Following Preacher et al. [78], bootstrapping procedures was used by drawing 5000 random sample to determine the significance of the mediation hypothesis (PROCESS Model 4). In addition, to test the moderated mediation hypothesis, this current study followed Preacher et al.’s [78] guidelines to construct bias-corrected confidence intervals at various levels of job autonomy using bootstrapping procedures (PROCESS Model 7).

### 3.2. Hypotheses Testing

Hypothesis 1 proposed that coworker support mediates the positive relationship between leader behavioral integrity and in-role autonomy. Empirical results demonstrate that the indirect effect of leader behavioral integrity on in-role autonomy is positive and statically meaningful (indirect effect = 0.14, SE = 0.05, bootstrap 95% confidence interval, CI: [0.057, 0.247]) because the CI did not include zero. Thus, Hypothesis 1 was supported.

Furthermore, it was tested whether job autonomy moderates the indirect effect of leader behavioral integrity on in-role autonomy via coworker support (Hypothesis 2). This current study found support for the conditional indirect effect of leader behavioral integrity on in-role performance. The indirect effect of leader behavioral integrity on in-role performance via coworker support is positive when job autonomy is high (indirect effect = 0.15, SE = 0.06, CI: [0.057. 0.291]). When job autonomy is low, the indirect effect of leader behavioral integrity on in-role performance via coworker support is also positive (indirect effect =.09, SE = 0.05, CI: [0.033, 0.190]) but weaker than when job autonomy is high. The index of moderated mediation is also significant (index = 0.03, SE = 0.02, CI: [0.006, 0.090]). This is consistent with the argument that leader behavioral integrity is more important for employees who perceive high job autonomy by helping them receive more coworker support which, in turn, impacts their in-role performance. 

## 4. Discussion

This study explores the underlying mechanism and boundary condition that account for why and under what circumstances leader behavioral integrity leads to an employee’s high in-role performance. The primary purpose of this study was to empirically examine the effect of leader behavioral integrity on an employee’s in-role performance via its impact on a focal employee’s coworkers. This study confirmed the mediating role of coworker support in the relationship between leader behavioral integrity and in-role performance. Given the importance of coworkers in the workplace especially in contributing to an employee’s effectiveness outcomes (e.g., [39,40,41,43]), the results showed that leader behavioral integrity can influence an employee’s behaviors via facilitating coworkers’ supportive behaviors. Furthermore, the present study represents one of the first attempts to examine the moderating effects of job characteristics in explaining the impacts of leader behavioral integrity. This present study found that compared to those who perceive low job autonomy, employees who perceive high job autonomy receive more coworker support resulting from leader behavioral integrity, which energizes their in-role performance. 

### 4.1. Implications for Theory and Practice

This study contributes to the leader behavioral integrity literature in two ways. First, while answering the calls for illuminating the underlying mechanism between leader behavioral integrity and work outcomes [21], this present study advances the leader behavioral integrity theory by introducing and examining the mediating role of an employee’s coworkers in explaining the leader behavioral integrity and in-role performance link. Past studies have established the mediating roles of trust in leadership (e.g., [12,14]) and role clarity resulting from clear communication between an employee and a manager (e.g., [12,14]). These prior studies suggest that leader behavioral integrity drives employees’ positive attitudes and perceptions toward their supervisor and role expectation in the dyadic relationship between an employee and a supervisor. In contrast, the current study suggests that team members’ supporting behaviors resulting from leader behavioral integrity plays an important role in explaining the leader behavioral integrity and in-role performance link. It is also noteworthy that with the except of a few studies [15,32], there is a dearth of research examining the impacts of leader behavioral integrity on team members. The results from this current study, therefore, provide new insight into a novel mechanism through how leader behavioral integrity leads to high in-role performance.

More broadly, this study also contributes to leadership research on leader traits. Past study has focused on leader behaviors [78,79,80,81,82] and the employee’s perceptions toward a leader [83] and work attitudes [84] as key mediators in the relationship between leader traits and various outcomes, such as leader effectiveness and emergence and employees’ performance. Accordingly, how leader traits affect team members behaviors and not just a single employee’s behaviors or attitudes, received little attention from leadership scholars. As such, the results from this current study expand the understanding of multiple mechanisms through how leader traits affect employee outcomes.

Second, this study contributes to the leader behavioral integrity literature by examining job autonomy as an important moderator in the relationship between leader behavioral integrity and in-role performance. Specifically, given the scarcity of research on the roles of perceptual moderators [23] and calls for research about the moderating roles of autonomy in in explaining the effects of leader behavioral integrity [27], the results from this present study expand the current literature on leader behavioral integrity by demonstrating that leader behavioral integrity can lead to higher in-role performance via coworker support especially when an employee perceives high level of job autonomy. Given that scholars have paid scant attention to the boundary conditions for the effects of leader behavioral integrity, this study offers novel insights to the boundary conditions where leader behavioral integrity can bring more positive impacts on an employee’s in-role performance. 

The current study also expands the literature on the JD-R model by considering the relationship between job resources at task-level (i.e., job autonomy) and interpersonal-level (i.e., coworker support). Most prior research on job characteristics model as well as JD-R model focused on the direct effects of job resources on outcomes of interest [85,86,87]. On the other hand, the results from this present paper highlight how one type of job resource can increase the other type of job resources which, in tandem, increases an employee’s in-role performance. Specifically, the current study suggests that leader traits (i.e., leader behavioral integrity) and job autonomy interact in explaining coworker support. Therefore, more broadly, these results contribute to the research stream which proposes the interaction effects of leader’s individual differences and job resources in predicting an individual’s outcomes [88,89,90].

From a practical perspective, this current research offers important implications for organizations. Employees’ cooperativeness is integral for team performance as well as team information-sharing [91]. The results suggest that the word-deed alignment of managers is integral in facilitating cooperation within a team because employees who perceive high leader behavioral integrity are motivated to support their leaders by reciprocating supporting behaviors toward their coworkers [8]. Thus, organizations can use leader behavioral integrity as the basis for leader selection as well as leader assessment especially in situations where leaders must manage teams where cooperativeness among its members is the key for team effectiveness.

The results also suggest that job autonomy enhances the positive effects of leader behavioral integrity on coworker support and in-role performance of employees. This is consistent with findings from the personality literature which shows that managers’ extraversion and conscientiousness were stronger predictors of their job performance when managers were in jobs with high autonomy [88]. In other words, organizations that assigned more job autonomy to its managers allowed the positive traits of these managers to improve the performance of the team that they manage. Thus, following the advice of Hackman and Oldham [45], organizations may consider redesigning jobs for employees to allow for more autonomy so that these employees may more freely act on their supervisor’s clearly directives and feedback and seek the needed support from their team members to perform their tasks. Since job autonomy largely consists of autonomy (1) in selecting methods to do one’s job, (2) in scheduling the tasks to perform the job, and (3) in selecting how one is evaluated on the job [73], organizations may redesign employees’ roles in teams to allow for more autonomy in one or more of the three areas in dictating how they perform their roles. This present study shows that by doing so, organizations may see the greatest gains in workgroup cohesiveness and in-role performance of employees who are under the supervision of a leader with high behavioral integrity.

### 4.2. Limitation and Future Research Directions

This study is not without limitations. First, the theory surrounding the moderating role of job autonomy rests on the assumption that employees who perceived a high level of job autonomy are more likely to ask for help or job-relevant information from their coworkers. It is based on past research findings that job autonomy increases opportunities to seek out more job-relevant resources from others [52,53]. However, as employees’ propensity to ask for help from coworkers is not measured in this study, future research would benefit from empirically testing the assumption by incorporating, for example, an employee’s proactive personality or information-seeking behaviors. 

Second, the model of this current study suggests that the positive effects of leader behavioral integrity on an employee’s in-role performance via perceived coworker support occurs at the individual level. However, given the importance of cooperation for group effectiveness and leader effectiveness [92], future research can further advance the findings from this current study by examining the model of this current study at the team-level. Moreover, considering the dearth of leader behavioral integrity research conducted at the team-level [13,32], investigating the effects of leader behavioral integrity on team-level outcomes could expand the current literature on leader behavioral integrity.

Third, this paper examined the moderating effect of job autonomy in the proposed mediation process. However, the authors acknowledge that other moderators may affect the relationship qualities at work and could further explaining the underlying mechanisms between leader behavioral integrity and in-role performance. For instance, future research could explore the moderating roles of employee–team relationship, such as team identification [93], or relationships among team member, such as team–member exchange [94], because these may influence employees’ prosocial behaviors towards their team members.

Fourth, although data was collected from multiple sources using unique employee–manager dyads, the research design of this current study was cross-sectional. Thus, this paper cannot rule out the possibility that the cross-sectional research design might bias the results from this study due to its preclusion of causal inference [95,96]. Future research should constructively replicate the model of this current research using a longitudinal study design for more rigorous findings.

Lastly, this current study was conducted in South Korea. Given that most previous studies on behavioral integrity were conducted in western countries, this study provides valuable contribution to the literature. However, the authors acknowledge that the extent to which the word-deed alignment is perceived valuable by employees can be different across national cultures [13,97]. For example, while some cultures define appropriate behaviors based on the characteristics of relationships, others focus more on the formal rules or contracts [98]. Thus, future studies involving employees in nations with different cultures would be beneficial in validating and increasing the generalizability of the findings from this study.

## 5. Conclusions

This study highlights the underlying mechanisms through how leader behavioral integrity enhances employees’ in-role performance. Specifically, leader behavioral integrity facilitates cooperative behaviors within a team which, in turn, allows employees to possess adequate resources to better perform their jobs. In addition, the extent to which leader behavioral integrity is translated into in-role performance via coworker support is moderated by the level of perceived job autonomy. That is, leader behavioral integrity could benefit employees more by allowing them have more latitudes at work. Thus, to benefit more from leader behavioral integrity, organizations may consider redesigning jobs to allow more autonomy to employees.

## Figures and Tables

**Table 1:** Descriptive statistics.

Variables	Mean	S.D.	1	2	3	4	5
1. Leader–member exchange	4.68	0.88	(0.89)				
2. Leader behavioral integrity	5.13	0.90	0.17 *	(0.94)			
3. Coworker support	5.07	0.82	0.08	0.55 ***	(0.85)		
4. In-role performance	5.21	0.90	0.31 ***	0.27 ***	0.33 ***	(0.92)	
5. Job autonomy	4.86	0.91	0.16 *	0.33 ***	0.29 ***	0.02	(0.94)

*N* = 209. * *p* < 0.05. *** *p* < 0.001. Reliabilities are on the diagonal in parentheses.

**Table 2:** Hierarchical regression results.

Variables	Coworker Support				In-Role Performance
	Model 1	Model 2	Model 3	Model 4	Model 5
Leader–member exchange (control)	0.10	0.00	−0.01	0.00	0.30 ***
Leader behavioral integrity		0.55 ***	0.51 ***	0.51 ***	0.13 ***
Job autonomy			0.13	0.13 *	−0.15
Leader behavioral integrity x Job autonomy				0.13 *	−0.04
Coworker support					0.27 ***
Adjusted *R*^2^	0.01	0.30	0.31	0.32	0.20
Δ*R*^2^		0.30	0.01	0.02	
Overall *F*	1.93	44.46 ***	31.44 ***	25.31 *	11.35 ***
Δ*F*		86.18 ***	4.06 *	5.03 *	

*N* = 209. * *p* < 0.05. *** *p* < 0.001.
